# Advocating Electrically Conductive Scaffolds with Low Immunogenicity for Biomedical Applications: A Review

**DOI:** 10.3390/polym13193395

**Published:** 2021-10-02

**Authors:** Dania Adila Ahmad Ruzaidi, Mohd Muzamir Mahat, Saiful Arifin Shafiee, Zarif Mohamed Sofian, Awis Sukarni Mohmad Sabere, Rosmamuhamadani Ramli, Hazwanee Osman, Hairul Hisham Hamzah, Zaidah Zainal Ariffin, Kishor Kumar Sadasivuni

**Affiliations:** 1 Faculty of Applied Sciences, Universiti Teknologi MARA, Shah Alam 40450, Malaysia; dniahmad1998@gmail.com (D.A.A.R.); rosma614@uitm.edu.my (R.R.); 2 Kulliyyah of Science, International Islamic University Malaysia, Bandar Indera Mahkota, Kuantan 25200, Malaysia; sabs@iium.edu.my; 3 Department of Pharmaceutical Technology, Faculty of Pharmacy, Universiti Malaya, Kuala Lumpur 50603, Malaysia; ms_zarif@um.edu.my; 4 Kulliyyah of Pharmacy, International Islamic University Malaysia, Bandar Indera Mahkota, Kuantan 25200, Malaysia; awissabere@iium.edu.my; 5 Centre of Foundation Studies UiTM, Universiti Teknologi MARA (UiTM), Cawangan Selangor, Kampus Dengkil, Dengkil 43800, Malaysia; hazwanee@uitm.edu.my; 6 School of Chemical Sciences, Universiti Sains Malaysia (USM), Gelugor 11800, Malaysia; hishamhamzah@usm.my; 7 Center for Advanced Materials, Qatar University, Doha P.O. Box 2713, Qatar

**Keywords:** PEDOT: PSS, conducting polymer, conductive scaffolds, degradation rate, biocompatibility, fabrication of scaffolds, biomedical application, tissue engineering

## Abstract

Scaffolds support and promote the formation of new functional tissues through cellular interactions with living cells. Various types of scaffolds have found their way into biomedical science, particularly in tissue engineering. Scaffolds with a superior tissue regenerative capacity must be biocompatible and biodegradable, and must possess excellent functionality and bioactivity. The different polymers that are used in fabricating scaffolds can influence these parameters. Polysaccharide-based polymers, such as collagen and chitosan, exhibit exceptional biocompatibility and biodegradability, while the degradability of synthetic polymers can be improved using chemical modifications. However, these modifications require multiple steps of chemical reactions to be carried out, which could potentially compromise the end product’s biosafety. At present, conducting polymers, such as poly(3,4-ethylenedioxythiophene) poly(4-styrenesulfonate) (PEDOT: PSS), polyaniline, and polypyrrole, are often incorporated into matrix scaffolds to produce electrically conductive scaffold composites. However, this will reduce the biodegradability rate of scaffolds and, therefore, agitate their biocompatibility. This article discusses the current trends in fabricating electrically conductive scaffolds, and provides some insight regarding how their immunogenicity performance can be interlinked with their physical and biodegradability properties.

## 1. Introduction

Advances in tissue engineering (TE) promise novel techniques to accelerate the recovery of damaged tissues by overcoming autologous, allogeneic, and xenogeneic tissue repair [1]. The three essential elements of TE are cells, scaffolds, and growth factors [2]. The success rate of TE is dependent on the ability of porous 3D scaffolds to mimic the function of the extracellular matrix (ECM) of a specific tissue. Scaffolds should also provide a compatible environment for the regeneration of tissues and the transplantation of organs [1,3]. Implantable scaffolds can meet the required criteria of biocompatibility, porosity, cell viability, and mechanical properties [4] by ensuring: (1) a greater control over the scaffold’s surface topography, surface wettability and surface charge [5]; (2) an optimised scaffold’s mass transportability to facilitate nutrient exchange and waste removal; (3) optimised biocompatibility to minimise the risk of toxicity [6,7,8]; (4) the presence of biological cues for cell fate to induce tissue regeneration [9,10,11]; (5) similarity in the rate of scaffold biodegradation and tissue growth [12,13]. The continuous development of biocompatible and biomimetic scaffolds is paramount to realise its clinical applications in improving the patient’s care and quality of life [8,14].

**Figure 1:**
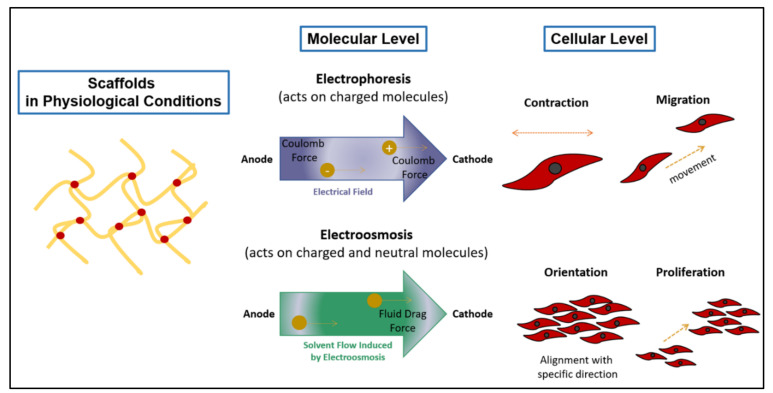
Illustration of the electrical conduction stimulation from conductive scaffolds, which enables cellular signalling and function in tissues.

Initially, scaffolds only act as a support system for cells to attach and proliferate. Due to this limited functionality, scaffold-based tissue engineering is primarily focused on improving tissue recovery by electrical stimuli through the application of conductive polymers (CPs). CPs exhibit electrical conductivity due to a reduction in their neutral state [15,16], and because of the presence of conjugated double bonds along the backbone. Dopant ions were commonly added into the CP’s chemical structure to neutralise the unstable backbone of the polymer in its oxidised state by donating or accepting electrons [17]. Poly (3,4-ethylene dioxythiophene): poly (4-styrene sulfonate) (PEDOT: PSS) is an example of a biocompatible CP that is commonly used to produce conductive scaffolds. In another study focusing on CPs, the electrical deterioration of scaffolds could be prevented by immobilising the dopant in a polyaniline (PANI) conductive scaffold patch. This was achieved as a result of the strong chelation bonding between a phytic acid dopant and chitosan [18]. In addition, several studies have demonstrated that conductive scaffolds with polypyrrole (PPy) can promote the regeneration of nerves, bones, muscles, and cardiac cells through electrical stimulation [19]. Figure 1 shows that the electrical conduction mechanism from conductive scaffolds enables cellular signalling and function in tissues to replicate normal electrophysiology; therefore, it causes electroactive cells to align in a specific direction, as well as to migrate and proliferate. Despite the advantages of using electroactive polymers in TE, the poor biodegradability of CPs is a barrier to realising their true potential [20,21,22,23].

The subject of biodegradability demands great attention in the pursuit of fabricating a conductive scaffold. The biodegradability of scaffolds is desirable, as it allows the materials to naturally dissipate, either via absorption or elimination in physiological conditions [24]. According to previous studies, conductive scaffolds were incorporated with natural compounds to make them biodegradable. Collagen and chitosan are examples of natural compounds that can physiologically degrade, without leaving behind any toxic residues. The application of non-toxic materials in scaffolds can minimise the adverse responses of the immune systems. However, studies on the immunogenicity performances of conductive and biodegradable scaffolds are scarce [5,12,14,25,26].

**Figure 2:**
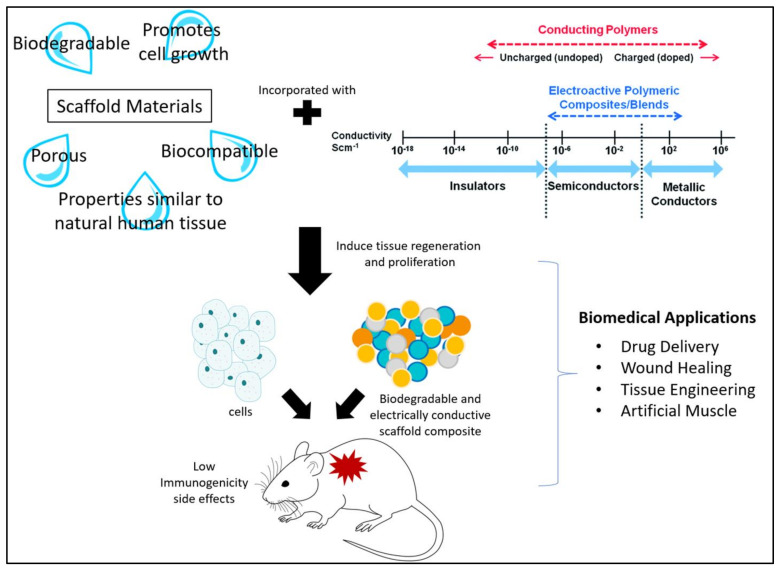
Illustration of the use of biodegradable and electrically conductive materials to promote cell growth with low immunogenicity.

It is vital to assess the efficiency of conductive scaffolds, in terms of their biodegradability and electrical conductivity. Introducing biomaterials into the human body, theoretically, triggers an immune response. However, a mild impact can be expected from a conductive-implantable scaffold with a stable biodegradation rate, compared to the scaffolds with an unstable biodegradation rate. Regardless, the by-products of the scaffold biodegradation process should be non-toxic to the host. Toxic by-products cause excessive dead cells and damaging tissues, thus compromising the functions of organs. Therefore, the use of safer biodegradable materials with non-toxic by-products is desirable for the growth and proliferation of the cell. Figure 2 illustrates that, with the use of suitable materials, we can ideally alter the immunogenicity of scaffolds.

Although organ transplantation is a proven method to address organ complications, this method is severely limited by the difficulties in securing replacement organs from donors [11]. Scaffold-based TE could be the key to this conundrum. This review article will discuss the recent fabrication techniques that can be employed to fabricate scaffolds. The contribution of the crosslinking process in regulating the degradation rate of scaffolds will be presented. A review of scaffolds’ physical properties, mainly regarding their mechanical and electrical conductivity performance, will also be discussed, according to their type of CP substituents. In addition, the biodegradation trends of conductive scaffolds in phosphate-buffered saline (PBS) will be reviewed, together with a juxtaposition with human immunogenic responses. Another feature of this field of research is that its outcomes can spur innovations in drug delivery processes [27]. In the latter part of this review, we outline several of the current developments of biodegradable natural materials and synthetic polymeric materials for various biomedical applications, including tissue engineering, wound healing, and drug delivery.

## 2. Techniques in Fabricating Electrically Conductive Scaffolds

**Figure 3:**
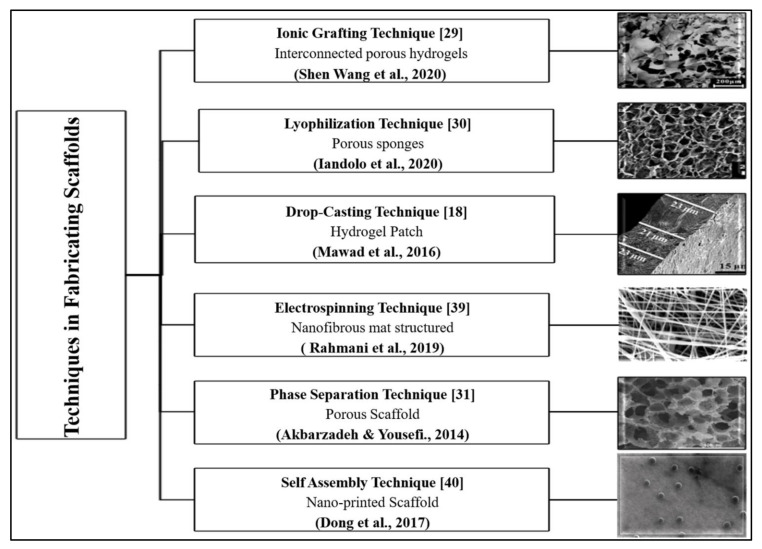
Techniques in fabricating scaffolds and their respective scanning electron microscope images [18,29,30,31,39,40].

**Figure 4:**
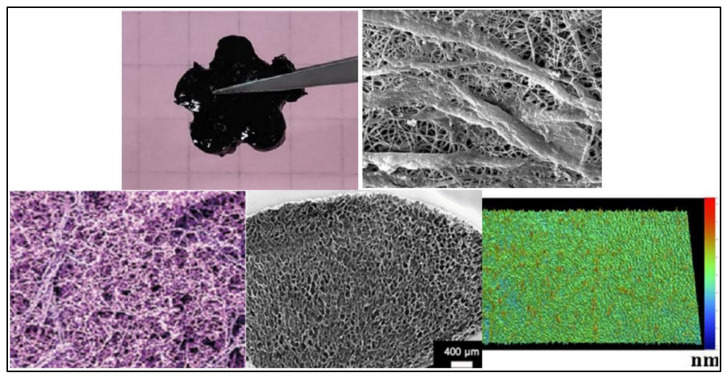
Images of scaffold structures: porous hydrogel, nanofibrous, porous hydrogel scaffold, porous sponge scaffold, and hydrogel patch scaffold [18,29,30,34,39].

The fabrication and the design of conductive scaffolds influence their performance in physiological conditions. Therefore, the required properties for a specific tissue should be identified before a scaffold is produced. The selection of materials for the scaffolds is essential to promote a safe environment for cell proliferation. Scaffolds can be fabricated from natural polymers, such as silk, collagen, keratin, cellulose, and chitin. Synthetic polymers, such as polylactic acid (PLA), polyglycolide acid (PGA), and polyhydroxyalkanoates (PHA) can also act as the base material of scaffolds. Figure 3 and Table 1 list the common techniques that are used to fabricate conductive scaffolds [17,24,28,29,30,31,32]. Additionally, Figure 4 shows that distinct scaffold fabrication techniques yield distinct forms and structures of scaffolds, including interconnected porous, hydrogel, and nanofibrous mat [18,25,29,30,33].

At present, electrospinning and lyophilisation are two standard techniques that are used to fabricate porous scaffolds [34,35]. The electrospinning method is used to obtain the nanofiber. Nanofibrous structured scaffolds can resemble ECM tissues on the nanoscopic scale [14]. In order to produce an electrically conductive nanofibrous scaffold, CPs are usually mixed into the scaffold matrix using physical blending or through coating techniques. The combination of CPs with spinnable polymers was found to facilitate the electrospinning process, while encouraging a micro- or nano-fibre structure. A composite-conductive scaffold can be realised by adding a layer of CPs onto electrospun fibres, during the combined process of electrospinning and spin coating [25,36,37,38]. However, a porous scaffold that is structurally similar to foams and sponges is more stable than a nanofibrous-structured scaffold created from electrospinning. The distribution of pores in a scaffold can be rearranged randomly or by following an organised pattern. The choice of pore distribution can be accomplished by manipulating the solvent and phase separating conditions during the scaffold fabrication process. The mechanical mismatch between the host tissue and the scaffolds can be minimised by applying the best scaffold fabrication technique for the target tissue. Minimising the discrepancies in mechanical compatibility is essential to encourage the host tissue’s acceptance of foreign scaffolds.

A three-dimensional (3D) composite with a high concentration of PEDOT: PSS-gelatine-bioactive glass scaffold was fabricated with the freeze-drying method to promote cell response, attachment, and viability. These advantages increase the number of functional human mesenchymal stem cells (hMSC) [41]. Shen Wang et al. (2020) fabricated interconnected porous hydrogel scaffolds with an ionic grafting technique. In this technique, the conductive PPy polymer was grafted onto methacrylic anhydride gelatine in the presence of ferric ions. The reversible ionic interactions between ferric ions, the gelatine, and PPy mixtures afforded the hydrogels with self-healing abilities, injectable capabilities, and high electrical conductivity [29]. In addition, the incorporation of gelatine in scaffold composites introduced injectable capabilities, which is a valuable feature for nerve tissue regeneration. Although the biodegradation processes did reduce the electrical conductivity of the scaffolds, the scaffolds still met the minimum requirement of the electrical stimulation characteristic for application in neural TE [42,43]. A strong understanding of the interaction between cells and conductive materials is indispensable in researching smart biomaterials for their application in TE. Recently, electrically conductive hydrogels have been fabricated and designed using a 3D printing method, which includes fused deposition modelling (FDM), direct ink writing (DIW), inkjet printing, and stereolithography (SLA) methods [44]. For example, conductive polymer carbon nanocomposites as the main thermoplastic filament materials for the FDM method have been employed as emerging electrochemical sensing devices [45]. However, the main drawback of 3D printing methods is the low printing resolution that is produced at the end of the fabrication process.

Mawad et al. (2016) reported that the electrical stability of the PANI scaffold patch could be improved by immobilising the dopant, phytic acid, together with a PANI on the surface of a biocompatible chitosan film [18]. The PANI patch scaffold was prepared by drop-casting chitosan solution onto a glass slide, followed by drop-casting a mixture of aniline, phytic acid, and ammonium persulfate onto the chitosan film. The ability of the scaffold patch to sustain its conductive state under physiological conditions can instigate an electronic interface between the biomaterials and the implantation site. A dopant is typically introduced during the synthesis of CPs, to produce favourable electrical characteristics. Dimethyl sulfoxide (DMSO), ethylene glycol (EG), sulfuric acid (H_2_SO_4_), phytic acid, and *p*-Toluenesulfonic acid (*p*TSA) are commonly employed as dopants [46,47,48,49,50,51,52,53,54]. In their research of crosslinked porous 3D hydrogel–PEDOT CP scaffolds, Mawad et al. (2016) mixed all of the required chemicals, followed by a filtration process [55]. It was found that the electroactive region of the PEDOT hydrogel network was covalently bonded to a hydrophilic polymer, and could be further altered to fabricate a covalently linked polymeric network.

Recently, the antibacterial, mechanical, and physical properties of a collagen-chitosan sponge scaffold, constructed from aquatic sources, were studied [56]. The researcher applied the lyophilisation technique (−50 °C, 0.5 Mbar vacuum pressure) to acquire a highly porous-structured scaffold. Furthermore, they added an alginate compound to the scaffold to improve its mechanical properties. The sponge scaffold’s porosity was in the range of 88–95%, with the addition of biopolymers, such as alginate and chitosan, onto the collagen [56]. Moreover, injectable-electroconductive hydrogels have the potential to improve cell survival, which could be translated into a novel treatment protocol [29,42,57], while minimising the need for invasive surgery. Additionally, the incorporation of electroconductive nanomaterials in hydrogels may influence their bulk electrical properties and topography, which can also affect the retention and biology of living cells. For instance, the application of CPs, such as PEDOT: PSS, in hydrogel scaffolds promotes the rhythmic beating in a neonatal rat cellular matrix [34,58]. The use of PEDOT in chitosan-based gels also resulted in an excellent regenerative capacity [43].

Iandolo et al. (2020) studied biomimetic scaffolds that supported neural crest-derived stem cell osteogenic differentiation [30]. The highly porous scaffolds were prepared by combining PEDOT: PSS with collagen type I, using the ice-templated technique. Collagen type I and PEDOT: PSS were blended in an ultrasonic bath to ensure the mixture’s homogeneity. Next, the mixture was poured into a specific mould with the desired shape, followed by freeze-drying and thermal treatment. (3-glycidyloxypropyl) trimethoxysilane (GOPS) was used as the crosslinker to enhance the mechanical properties of the scaffolds. Heat treatment was performed to reticulate the crosslink [30]. The outcome of this study was a porous scaffold with the desired geometry. The ice templating process was necessary to create highly porous scaffolds that were compatible with cell infiltration and proliferation.

Another technique that is used to fabricate conductive scaffolds is electrospinning. Abedi et al. (2019) studied the fabrication and characterisation of conductive nanofibrous chitosan/PEDOT: PSS for cardiac TE. PEDOT: PSS was paired with chitosan due to its chemical and thermal stability. The combination promoted the scaffold’s electrical properties, mirroring the myocardium ECM. A double nozzle electrospinning apparatus was used to form a fibrous mat structure of a conductive scaffold. The scaffold was then crosslinked using the glutaraldehyde solution vapor technique [25]. They discovered that the addition of PEDOT: PSS into chitosan/polyvinyl alcohol (PVA) increased the scaffold’s conductivity. The electrospinning and crosslinking of the fibrous mat scaffold increased its mechanical strength. The presence of hydrogenic bonds, between OH groups in PVA and chitosan, and SO^−3^ groups in PSS in the CPs’ dispersion, also contributed to its higher mechanical strength. Interestingly, the fibrous mat of the chitosan/PVA/PEDOT: PSS scaffold promoted the attachment and proliferation of cells.

When fabricating and designing a suitable and safe TE product, scaffolds should be able to biodegrade and should support cellular growth in vitro and in vivo. However, other aspects, such as attachment, migration, and cell proliferation, must also be taken into consideration. These aspects are highly dependent on the scaffold’s surface properties and its interaction with transmembrane proteins. Low-risk tissue regeneration processes can be achieved with advanced scaffold technology.

## 3. The Crosslinking Process in Fabricating Conductive-Polymeric Scaffolds

**Figure 5:**
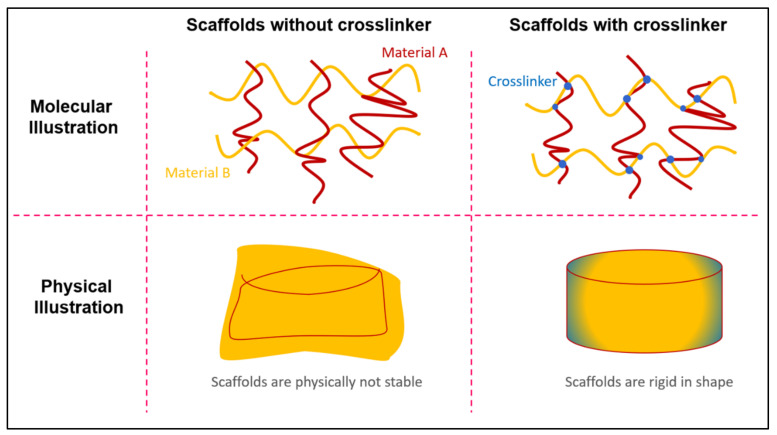
Illustration of the enhancement of a scaffold’s mechanical properties by employing the crosslinking processes.

During the fabrication of polymeric scaffolds, the crosslinking process is adopted to chemically bind the molecules within the scaffold’s structure. This, in turn, significantly influences their mechanical properties. For example, the crosslinking process upon a nanofibrous-structured scaffold causes the fibres to attach and stack between each other, resulting in better resistance performance to the application of shearing forces. The crosslinker can be divided into three types: chemical crosslinker, biophysical crosslinker, and enzymatic crosslinker. Adding crosslinkers can enhance their mechanical properties; however, this can affect their physical condition, causing the scaffolds to become contracted or shrunk. However, in the presence of dehydrothermal (DHT), crosslinking can prevent scaffold contraction, thus making scaffolds more stable for a long period of time. Meanwhile, crosslinking with ultraviolet (UV) light can enhance the scaffold’s stability in an aqueous environment, and does not cause any physical changes to the scaffolds [58,59,60]. This may be due to the fact that the UV light crosslinking process does not take place chemically, and interacts with the compound that is present in the scaffolds. UV crosslinking hardened the liquid polymeric material to be more stable and to possess a rigid shape, with no heat exposure exerted on the material [61]. In addition, crosslinking can also avoid the premature dissolution of the scaffold at the normal body temperature [62]. Figure 5 shows an illustration of the mechanism of the scaffold’s crosslinking processes. Extensive research has been performed to develop biomedical scaffolds that meet the criteria of cost-effective fabrication, ease of customisation, and safe application in clinical settings, particularly in the areas of infection treatment and drug delivery [38,63].

The intermolecular crosslinking of collagen-based scaffolds, either through chemical or physical methods, can modify their mechanical properties [12,56]. However, some well-known crosslinking agents, such as glutaraldehyde (GTA), glyoxal, and glycol diglycidyl ether, are toxic [64,65]. According to Hua et al. (2020), sodium tripolyphosphate (TPP) is a safe crosslinker for collagen and chitosan, with excellent biocompatibility [66]. A comparative study of crosslinkers reported that genipin (GP) and TPP had better biocompatibility, compared to GTA, towards the collagen/chitosan scaffold. Nonetheless, TPP was recommended as the most suitable crosslinking agent for myocardial TE [67]. In their study, collagen and chitosan were dissolved in a 1% (*v*/*v*) acetic acid solution at room temperature. After fabrication was carried out using the lyophilisation method (−20 °C for 48 h), a 1:1 ratio of a lyophilised collagen/chitosan scaffold was crosslinked. In the case of collagen, crosslinking could be achieved through the formation of an amide, from the activation of the carboxylic group with the amine. In comparison, 1-(3-dimethyl aminopropyl)-3-ethyl carbodiimide hydrochloride and N-hydroxysuccinimide were employed as crosslinkers to strengthen the covalent attachment between the carbonyl group of gelatine and the amino group of chitosan in the porous PEDOT/Chitosan/Gel scaffolds [41].

Interestingly, a study focusing on poly(vinyl) alcohol-gellan gum-based nanofiber proved that a scaffold’s degradation rate could be controlled by various crosslinking agents [38]. In addition, for electroconductive hydrogels (EH), there are several types of suitable crosslinking processes, such as physical crosslinking, covalent crosslinking, and supramolecular crosslinking. However, each of these crosslinking techniques may affect the biodegradability of the EH scaffold. The density and concentration of the crosslinker can alter the properties of the scaffolds, including their water content, mesh size, porosity, diffusivity, and mechanical characteristics [60,68,69,70]. Overall, the use of crosslinkers could physically change the properties of scaffolds, which can affect the biodegradability of the scaffolds. This idea warrants further investigation, focusing on the execution of scaffolds’ optimum degradation rates by varying the concentration of any crosslinker.

## 4. Physical Properties of Conductive-Polymeric Scaffolds

No single polymeric system can be considered the ideal biomaterial for all medical applications, due to the complexity of the human body and the scope of applications that polymeric biomaterials are currently utilized for. Thus, a mixture of natural and synthetic polymers can overcome the limitations of a monocomponent system [12]. Theoretically, a composite scaffold that is composed of more than two sub-materials exhibits a greater characteristic value, due to the combination of materials that hold some desired properties. Due to the various needs of scaffolds, composite materials with excellent properties are commonplace in TE [71]. For example, Lari et al. (2016) fabricated nanohydroxyapatite/chitosan (nHAp/CS) composite scaffolds that were embedded with PEDOT: PSS through a lyophilization technique. They found that nHAp and PEDOT: PSS were homogeneously dispersed in the chitosan matrix. The CS/nHAp/PEDOT: PSS scaffolds exhibited a high cell attachment rate, due to their surface roughness. The electrical conductivity that was recorded for the CS/nHAp/PEDOT: PSS sample was 9.72 ± 0.78 µS/m. They also found that both the compressive modulus and the yield strength of the chitosan increased to 0.5–1.0 MPa and 5–5.5 MPa, respectively, with the addition of nHAp and PEDOT: PSS. Unfortunately, the CS/nHAp/PEDOT: PSS had poor biodegradability, due to the strong bonding between PEDOT: PSS and the rest of the substrate chains [26]. Meanwhile, Iandolo et al. (2020) prepared highly porous biomimetic scaffolds by combining the PEDOT: PSS with collagen type I (the most abundant protein in bone) for inactive support. However, aggregation took place when collagen was a part of the mixture, due to the interaction between the positively charged protein chains and the negatively charged polystyrene sulfonic acid groups, which caused the restructuring of the CPs. Unfortunately, this process contributed to the de-doping of the conductive PEDOT segments.

Abedi et al. (2019) demonstrated that the addition of PEDOT: PSS in chitosan scaffolds could improve their biocompatibility, cell viability, and mechanical and electrical properties. In their study, the electrical conductivity, elongation at break (%), ultimate strength, and toughness of CS/PVA/PEDOT: PSS scaffolds were recorded at 7.63 × 10^−3^ S/m, 5.6 ± 0.3%, 18.78 ± 0.95 MPa, and 48.87 × 10^6^ J m^−3^, respectively. They also discovered that the attachment length of the cells increased as the nanofiber diameter was decreased to 40 nm. Further shrinkage of the fibre diameter to less than 40 nm affected the attachment of cells. However, the addition of PEDOT: PSS can be toxic, depending on the weight composition. The addition of more than 0.6 wt.% PEDOT: PSS in gelatine scaffolds causes toxicity in stem cells; this means that the gelatine amount must be lower than 0.6 wt.% in a single scaffold composition [25]. Overall, previous studies established that PEDOT: PSS, which is favourable for cell proliferation, can be the predecessor for a highly biomimetic, electroactive scaffold for stem cell expansion and differentiation [30].

It is essential to establish a link between materials degradation in vitro and mechano-morphological characteristics, since biodegradation can negatively affect the mechanical and structural integrity of the scaffolds. Biodegradation is manifested by material loss in grafted scaffold structures because of ageing in the PBS medium. The biodegradation rate of a scaffold can be determined by the calculation of weight loss in PBS conditions. A higher percentage of mass loss in the scaffolds can be attributed to the high surface area and porosity of scaffolds [72]. For example, Zhang et al. (2008) investigated the in vitro biodegradation of an electrospun tubular protein scaffold, by immersing the scaffold in a PBS condition (pH = 7.3) for various timeframes. This was then followed by determining the scaffolds’ weight loss percentage. Ideally, the degradation rate of the scaffold should be similar to the rate of new tissue formation. The mechanical and electrical properties of various scaffolds are summarised in Table 2. In addition, the conductivity of the fabricated scaffold should refer to the sheet conductivity of the material, which is different from the commonly evaluated electrochemical impedance responses of the coated electrodes.

## 5. Biodegradation Mechanisms of Polymeric-Based Scaffolds

**Figure 6:**
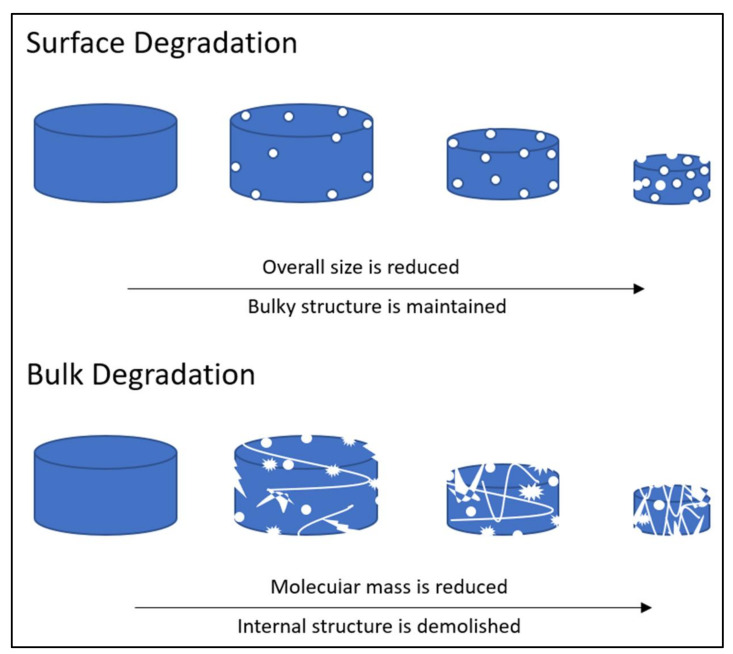
Illustrations of the surface and bulk degradation process.

Recent advances in biodegradable biomaterial synthesis have been directed toward developing and synthesizing polymers with properties that are tailored for specific biomedical applications. Understanding the biodegradation mechanism of scaffolds in the physiological environment is necessary to optimise their functionality [73]. There are two types of biodegradation process: surface degradation and bulk degradation. As illustrated in Figure 6, the type of biodegradation depends upon the diffusivity of water inside the matrix, the degradation rate of the polymer’s functional groups, and the size of the matrix. Polymeric scaffolds that experience surface degradation will preserve their bulky structures, even when their overall size is reduced. The bulk degradation of polymeric material will demolish the scaffold’s internal structure and reduce its molecular mass [74].

**Figure 7:**
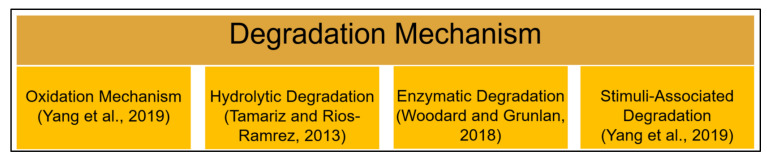
Different types of degradation mechanisms in physiological condition.

Biodegradable scaffold materials will experience a gradual breakdown that is dependent on biological, chemical, and biophysical aspects and factors. The four types of polymeric in vivo degradation mechanisms are hydrolytic, oxidation, stimuli-associated, and enzymatic [6,27,75,76], as summarized in Figure 7. The biodegradation of polymeric biomaterials such as scaffolds involves a hydrolysis process that is initiated by water molecules and the disintegration of sensitive bonds in the polymer by enzymes. These events cause the erosion of the polymer. The biodegradation rate depends upon the physiological environment and the intrinsic properties of the scaffolds, as follows: (1) the chemical structure, (2) the presence of hydrolytically unstable bonds, (3) the level of hydrophilicity and hydrophobicity, (4) the crystalline morphology, (5) the glass transition temperature, (6) the copolymer ratio, (7) molecular weight, (8) tacticity, (9) loading direction, (10) pH, and (11) the treatment processes that are involved during scaffold fabrication, such as crosslinking process [69,77].

**Figure 8:**
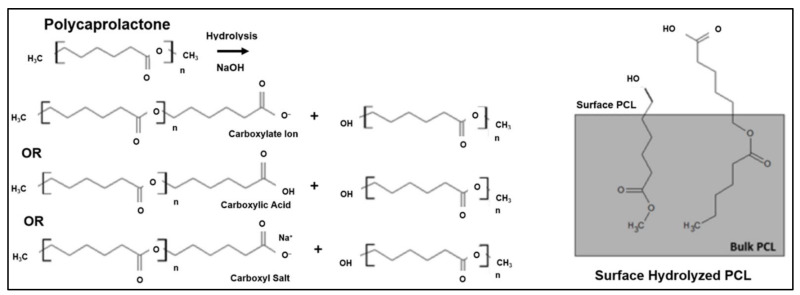
The hydrolytic degradation mechanism of PCL scaffolds [79].

The hydrolytic degradation of polymers entails the collapse of chemical bonds in the polymer backbone by water molecules. Acids, bases, or salts catalyse the degradation to form oligomers and monomers. This form of degradation significantly reduces the molecular weight of the polymer. A previous study reported that the addition of PPy causes the polymer chains in scaffolds to aggregate and resist water diffusion, which slows down the degradation rate. This is due to the increasing number of hydrophobic bonds that are present after the blending of PPy with the PCL-CS mixture [78]. Figure 8 is an example that illustrates the hydrolytic degradation mechanism of PLA scaffolds. Mild hydrolysis results in the slight degradation of the polymer’s surface, revealing the surface carboxyl and hydroxyl groups. Carboxyl may be present in several forms, including the carboxylate ion, carboxylic acid, and carboxyl salt [79].

**Figure 9:**
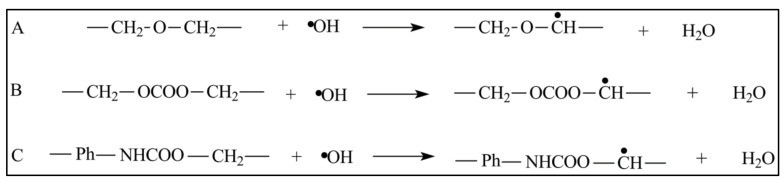
A mechanism of oxidative degradation by H_2_O_2_ in poly(ether urethanes) (**A**), poly(carbonate urethanes) (**B**), and aromatic polyurethanes (**C**) [80].

The degradation mechanism process can also be mediated by biological agents, such as enzymes, that partake in tissue remodelling. Polymeric scaffolds are also vulnerable to oxidation mechanisms. When scaffolds are exposed to body fluids and tissues, the host’s immune cells will initiate inflammatory responses. This situation can cause the release of highly reactive oxygenic molecules, such as hydrogen peroxide (H_2_O_2_), superoxide (O_2_-), nitric oxide (NO), and hypochlorous acid (HOCl). These molecules accelerate polymer chain scission and the degradation of scaffolds. Figure 9 shows an example of the oxidative degradation of poly (urethane) derivatives by hydrogen peroxide compound, including (A) poly(ether urethanes), (B) poly(carbonate urethanes), and (C) aromatic polyurethanes, which produce glycol ether radicals as by-products.

**Figure 10:**
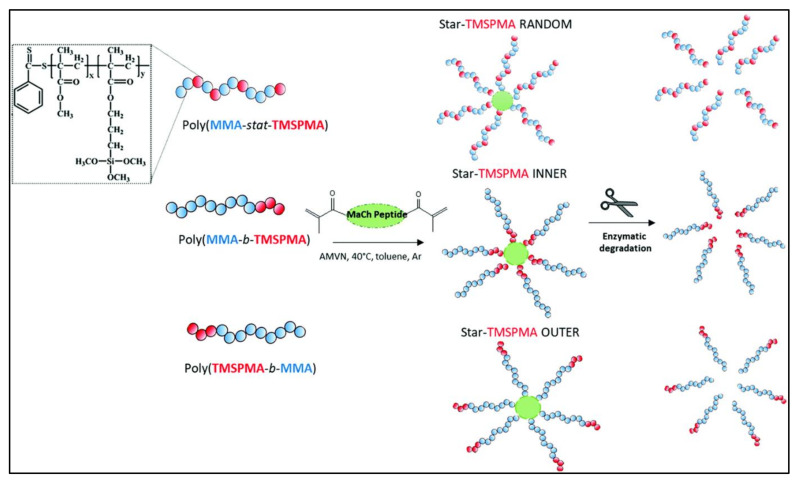
Illustration of the enzymatic degradation process [81].

The degradation mechanism process can also be mediated by biological agents, such as enzymes, that partake in tissue remodelling [73]. Figure 10 shows an example of the enzymatic degradation mechanism of methyl methacrylate-poly 3-(trimethoxysilyl) propyl methacrylate (MMA-TMSPMA) star polymers that are synthesised with the arms of three different architectures (random, inner, and outer), crosslinked with a dimethacryloyl peptide (MaCh-peptide) core, and cleaved with collagenase activity [81]. The enzymatic mechanism often proceeds, concurrently, with hydrolytic degradation. The presence of hydrolases enzymes, such as proteases, esterases, glycosidases, and phosphatases, catalyses the hydrolysis reaction of biomaterial disintegration [27]. The interaction between the enzymes and the polymeric chains begins with the diffusion of specific enzymes on the polymer’s solid surface. This diffusion is followed by the enzyme–substrate complex formation, whereby the substrate causes a conformational or shape change of the enzyme–substrate complex. Catalysis of the hydrolysis reaction occurs, and the soluble by-products diffuse into body fluids [6].

**Figure 11:**
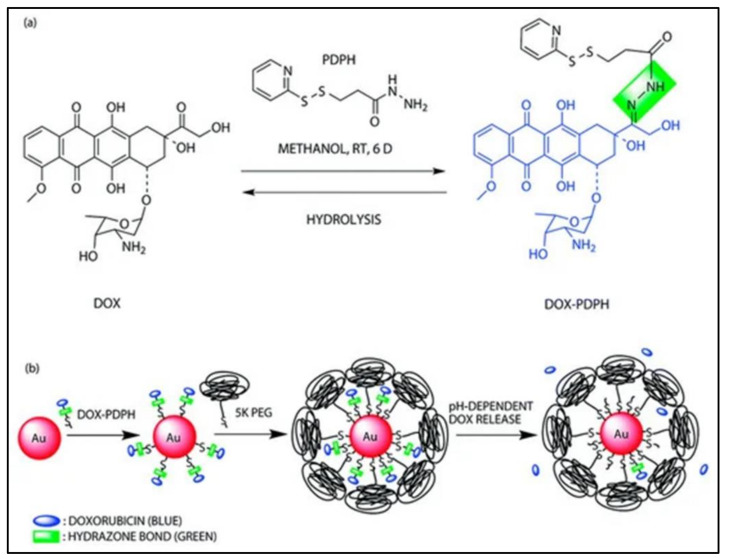
A pH-sensitive drug-gold nanoparticle system: (**a**) chemical synthesis of the doxorubicin–hydrazone linker conjugate (dox–PDPH); (**b**) schematic illustration of the synthesis of the multifunctional drug delivery system and its pH-dependent doxorubicin release [82].

Additionally, Figure 11 shows examples of the stimuli that are associated with degradation mechanisms; a pH-sensitive drug-gold nanoparticle system for tumour chemotherapy, and surface-enhanced Raman scattering (SERS) imaging [82]. This concept can be applied to scaffold degradation mechanisms with the use of doxorubicin drugs as fillers for a specific treatment. According to Yang et al. (2019), a stimuli-associated degradation mechanism that encourages scaffold swelling normally manifests in sol-gel degradation behaviour. In this case, the degradation process occurs by allowing the scaffold network structure to be cleaved by external triggers: pH-responsive, light-responsive and redox-responsive [8]. A pH-responsive smart hydrogel offers targeted and controlled release behaviour to wounds, while its network architecture remains intact, with slower degradation in normal tissues. A photo-responsive hydrogel goes through light-mediated degradation, while redox-responsive hydrogels react to internal and external oxidative and reductive stimuli.

The scaffold degradation rate should be on par with the tissue ingrowth to maximise healing or to deliver healable drugs. Generally, the degradation of polymers in physiological conditions is caused by a molecular chain scission that is initiated by hydrolysis (anhydride, ortho-ester, ester, urea, urethane/carbonate, and amide bonds) or enzyme-catalysed hydrolysis. A number of degradable polymeric scaffolds contain labile bonds that tend to hydrolyse. Additionally, these bonds are too stable under physiological conditions. Thus, they require an enzymatic catalyst to encourage degradation [70]. There are several non-invasive techniques to monitor in vivo scaffold degradation. Electron paramagnetic resonance (EPR) is an efficient and accurate technique to investigate radical and oxidative stresses [83]. Ultrasound elasticity imaging (UEI) can be used to characterise the structural, functional, and compositional changes of biodegradable scaffolds via phase-sensitive speckle tracking [84,85]. Several non-invasive and non-destructive techniques to investigate parameters such as a scaffold’s pH value, distribution, and cell viability are: (i) confocal laser scanning microscopy (CLSM); (ii) nuclear magnetic resonance (NMR); (iii) optical coherence microscopy (OCM); (iv) optical coherence tomography (OCT). OCT can be used in tandem with various light sources, such as near-infrared fluorescence (NIR) [86,87]. Zhang et al. (2020) innovated a multifunctional hydrogel system with tetraphenylethene (TPE), that has similar traits to aggregation-induced emission (AIE) nanoparticles, to monitor the degradation of hydrogel scaffolds in physiological conditions [88,89].

## 6. Immunogenic Effects on the Biodegradation Behaviour of Scaffolds

Immunogenicity is a biological response to the presence of non-compatible foreign substances or living organisms in the body. This reaction can cause complications for one’s health. Therefore, biocompatible scaffolds are imperative to circumvent immunogenic effects. The by-products of degradation, such as monomers, oligomers, and polymer fragments, should be biocompatible with the human body, and should be able to pass through filtering organs without causing any complications. Scaffold materials with a low degradation rate might lead to transplant failure, due to negative immune responses. Woodard and Grunlan (2018) [75] underlined the scaffold’s dimensions as a factor that influences degradation. This idea was also presented in another study [74].

**Figure 12:**
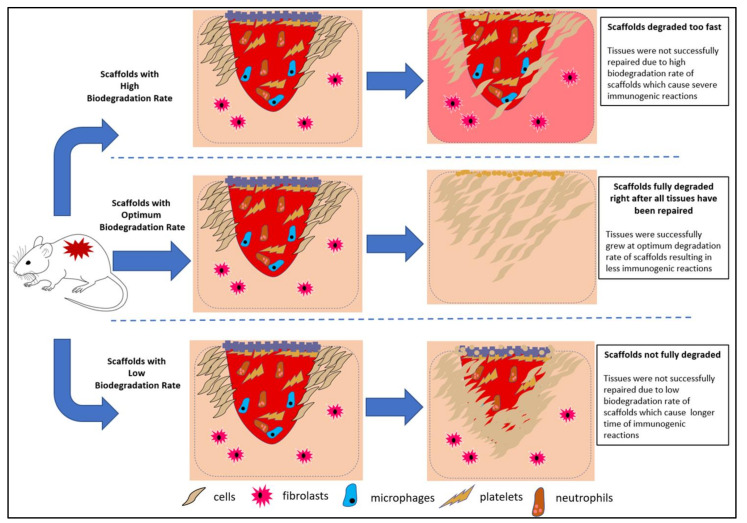
Illustration of the different immunogenic responses towards the specific biodegradation trends of scaffolds.

Figure 12 illustrates how different biodegradation rate trends are capable of tuning the immunogenicity of scaffolds. A poor degradation rate of a non-compatible scaffold will inhibit tissue regeneration, since the scaffold will continuously trigger immune reactions. However, incompatible scaffolds with high degradation rates could compromise tissue regeneration rates, due to the absence of cellular support. Altering the material degradation rate seems to be a promising means of optimising the scaffold’s biocompatibility. Natural polymers often exhibit better biocompatibility and biodegradability. However, the degradation of synthetic polymers can be tailored accordingly. Acidic by-products, which are hydrolytically produced from polyester scaffold degradation, may also cause physiological inflammatory responses [75]. In some cases, a low pH environment can unnecessarily accelerate the scission of scaffolds. Therefore, biodegradable polyester for physiological purposes can be optimised by introducing basic salts into the polyester, such as calcium carbonate, sodium bicarbonate, and calcium hydroxyapatite [75]. The use of biodegradable natural polymers, such as collagen, was suggested, according to the presence of collagenase as a physiological enzyme [90]. The presence of natural polymers in scaffolds only triggers mild biological responses, and this can prevent severe immunogenicity side effects.

## 7. Common Conductive and Biodegradable Scaffolds

### 7.1. PEDOT-Based Scaffolds

#### 7.1.1. Characteristics of PEDOT-Based Scaffolds

To utilize CPs for developing and synthesizing scaffolds with properties that are tailored for use in tissue engineering, the suitable conductive and hybrid systems of biocompatible scaffolds must be discussed. Extensive research is ongoing to optimise the stability of scaffolds, by incorporating composite materials to overcome the problems with tissues [18,38,58,68,91]. The scaffold materials must fulfil the criteria of convenient sterilisation, biocompatibility, and non-toxicity [92]. The seamless electrical communication among cells, and the optimised growth of cells, can be achieved by exploiting scaffolds with sufficient electrical conductivity. Recently, the focus of scaffold-based TE is on the enhancement of bone healing via electrical stimuli with CPs [40]. The addition of modified electroactive oligomers can control the biodegradability of CPs. These grafted copolymers are connected via degradable ester linkages, and are highly sought after in biomedical applications that employ pyrrole, aniline, or thiophene groups [23]. Unfortunately, electroactive aniline-based oligomers are toxic to human bodies. In response to this observation, Mawad et al. (2016) proposed 3,4-ethylene dioxythiophene (EDOT)-based oligomers as an alternative to replace aniline-based oligomers, to minimise the biomaterial’s toxicity [54].

**Figure 13:**
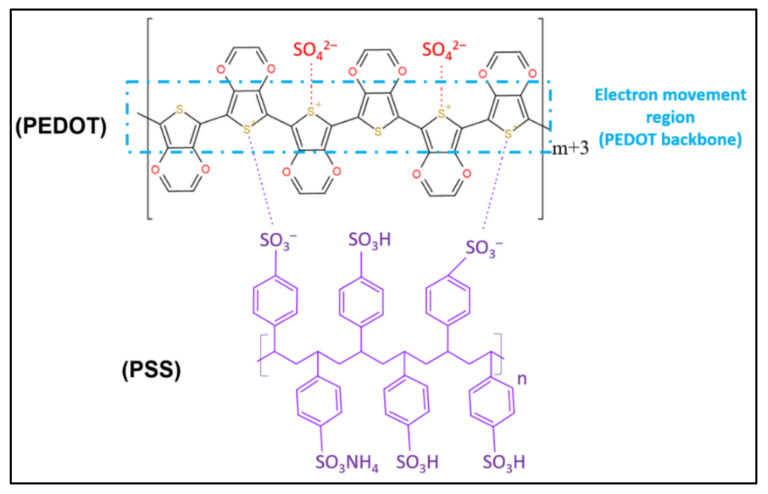
The chemical structure of PEDOT: PSS with the presence of sulfonate ions from an oxidant [94].

PEDOT can be chemically tuned to alter the mechanical and electrical properties of scaffolds. This tuning can promote the covalent attachment between biomolecules and scaffolds to become biocompatible [93]. In addition, conductive scaffolds are conductive for the differentiation and proliferation of electrically stimulated responsive cells [28]. PEDOT: PSS is a common biocompatible CP behind conductive scaffolds. Figure 13 shows the chemical structure of PEDOT: PSS. This copolymer has a moderate band gap and excellent stability [25]. For example, the addition of PEDOT: PSS in a chitosan-based electrospun scaffold not only enhances the scaffold’s mechanical and electrical conductivity, but also improves its biocompatibility and cell viability [24]. The findings regarding the biodegradability of various scaffolds including conductive scaffolds from previous research are summarised in Table 3. Predominant crosslinking in scaffold fabrication (as discussed in the Crosslinking Process section in Fabricating Conductive-Polymeric Scaffolds (Section 3)), increases the mechanical properties and stability of the scaffold at the possible expense of the scaffold’s biodegradability.

#### 7.1.2. Biodegradable Trends of Various Conductive PEDOT-Based Scaffold Composites

The research focusing on scaffolds for biomedical applications has made significant strides with the advent of new technologies. The mechanical and electrical properties of scaffolds will affect the scaffold’s biodegradation rate and biocompatibility [72,95,96,97]. Wang et al. (2017) experimented with varying weight percentages of PEDOT-HA nanoparticles in the fabrication of hyaluronic acid-doped PEDOT/chitosan/gelation porous conductive scaffolds [9]. They reported that the in vitro biodegradation of the scaffold had an inverse relationship with the weight percentage of PEDOT-HA. The scaffold displayed high biodegradability when there was a minute amount of PEDOT-HA. A large volume of PEDOT-HA enhanced the stability and biodegradation resistance of the scaffold. The addition of hydrophobic PEDOT likely reduced the hydrophilicity of the scaffolds [9].

Wang et al. (2017) also researched PEDOT nanoparticles/chitosan/gelatinous porous scaffolds. They claimed that the presence of PEDOT nanoparticles significantly reduced the degradation rate of the scaffolds. Interestingly, the presence of PEDOT nanoparticles in the scaffold increased the cell viability. This is due to the genial interactions between the scaffold and the cell surface, which encourage cell proliferation and growth [40]. Another contribution from Wang et al. (2018) was the study of PEDOT/chitosan/gelatinous scaffolds for neural cells. The electrical conductivity of hydrated and dehydrated PEDOT/chitosan/gel scaffolds gradually diminished over time [10]. This observation could be attributed to the disintegration of the PEDOT layers on the surface of the chitosan/gelatinous matrix. The dissipation of PEDOT layers occurs when chitosan and gelatine are gradually degraded in the presence of the enzymes that are supposed to stimulate physiological conditions. Although the electrical conductivity of the scaffold reduced over time, as summarised in Table 3, it still met the electrical conductivity requirements for electrical stimulation in neural TE application.

Another study demonstrated that the addition of conductive PEDOT: PSS in nHAp/chitosan composite scaffolds using the lyophilisation method reduced the scaffold’s biodegradability rate in the PBS solution. Although the scaffold with PEDOT: PSS had low biodegradability, its mechanical properties were consistent [26]. Lari et al. (2020) attempted to decrease the wettability, while dialling up the mechanical properties, of PEDOT: PSS/nHA/CS biocomposite, by integrating polycaprolactone (PCL) into the scaffold. PCL was selected due to its biodegradability and ease of blending with chitosan [5]. In another study, a PCL-CS-PPy conductive biocomposite nanofibrous scaffold is also a subject of interest in TE. The nanofibrous-structured scaffolds with sufficient biodegradability can be fashioned using electrospinning. It is worth mentioning that the mechanical properties decrease in tandem with the weight percentage of PPy. When the weight percentage of PPy used was reduced, it was easier for the scaffold to disintegrate, exhibiting the non-polymeric scaffold’s typical mechanical behaviour [68].

In addition, Abedi et al. (2019) fabricated a conductive nanofibrous chitosan/PEDOT: PSS scaffold using the electrospinning method [25]. They reported that the addition of PEDOT: PSS in the scaffold may support cell growth without any toxic effects. Nonetheless, the biodegradability of the scaffold has yet to be explored. It was reported that the use of PEDOT: PSS in fabricating the scaffold did not change the biodegradability of the scaffold, due to the presence of alginate [98]. In addition, they also stated that an increase in PEDOT: PSS concentration in gelatine-alginate scaffolds could increase cell proliferation, although they did not report the cause. A takeaway from these studies is that the addition of CPs in the polymer matrix is a prerequisite for acquiring electrically conductive scaffolds. Adjusting the weight percentage of the CPs in the matrix composite of the scaffolds will affect their biodegradability. Therefore, further studies focusing on the relationship between the addition of CPs and the superior biodegradability of conductive scaffolds are warranted.

### 7.2. Collagen-Based Scaffolds

**Figure 14:**
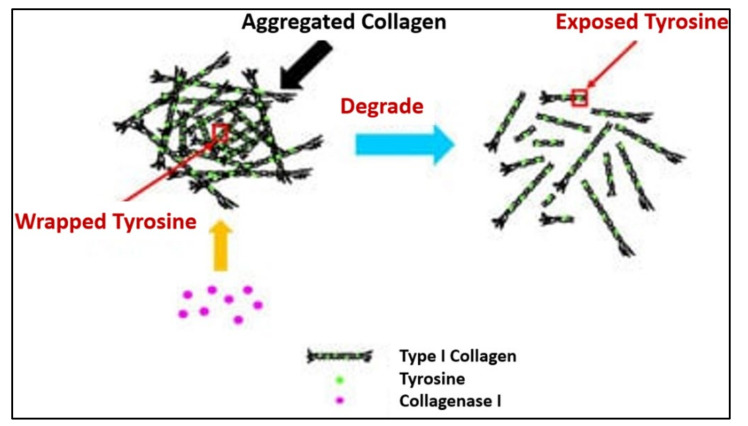
Process of collagen breakdown with the presence of a physiologic collagenase enzyme [100].

The addition of collagen or silk fibroin in scaffolds as biodegradable substituents can enhance their biodegradability, due to the nature of their proteins. Bioactive molecules, such as collagen, chitosan, and hydroxyapatite (HAp) were described as compatible, non-toxic, non-carcinogenic, non-immunogenic, and soluble in physiological conditions [13]. Due to these properties, the application of a collagen scaffold is prevalent in the field of damaged tissue regeneration [12,13]. Unfortunately, collagen is vulnerable to rapid degradation in body fluid or cell culture media [99]. The breakdown of collagen fibres depends upon the proteolytic action of collagenases, which are part of the large family of matrix metalloproteinases. For type I collagen, the cleavage site is specific, generating three-quarter and one-quarter length fragments. These fragments are further degraded by their matrix proteinases, as illustrated in Figure 14. Therefore, it is mandatory to blend collagen with other materials to augment the mechanical properties of collagen-based scaffolds.

### 7.3. Chitosan-Based Scaffolds

**Figure 15:**
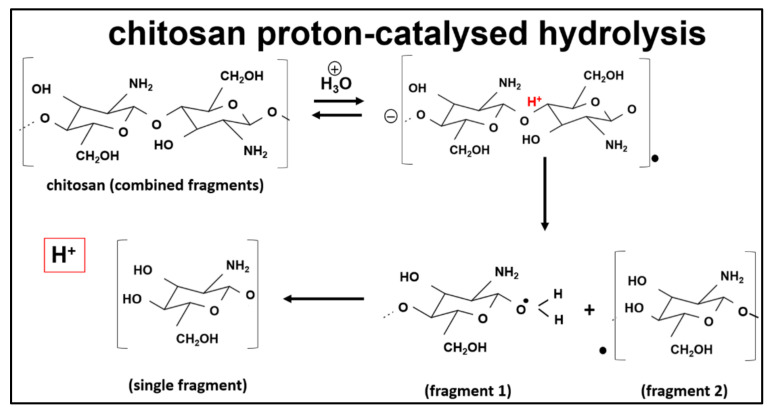
The breakdown of chitosan at pH 3 [76].

Another biodegradable polymer, chitosan, has become relevant in TE, due to its features and properties of low toxicity, non-immunogenic, and biodegradability, that are similar to the native ECM [5,9,14]. Chitosan degrades the body through physical and chemical degradation. The former entails swelling, cracking, and dissolution, while chemical degradation results from depolymerisation, oxidation, and hydrolysis [78]. Chitosan behaves as a hydrophilic cation, due to the electronegativity of its amino groups. The deacetylation degree (DA) of chitosan generally influences its polarity, pH, ionic strength and, ultimately, its water-soluble behaviour. Chitosan usually degrades at a pH below 6 [76]. The breakdown of chitosan through the pH degradation mechanism is illustrated in Figure 15. Chitosan is a polysaccharide with a cationic nature and displays outstanding properties, such as biocompatibility, hydrophilicity, and anti-thrombogenicity. Furthermore, chitosan can be combined with various polymer materials or drugs, using the appropriate preparation techniques [4,10,14,18,65,101,102,103]. Nonetheless, their mechanical and electrical attributes are unique, depending on their fabrication technique.

A study demonstrated that the addition of PEDOT: PSS into chitosan/PVA altered the mechanical properties and conductivity of the scaffold [24]. Conductive polymeric scaffolds with low degradation rates can be turned into composite scaffolds with high degradation rates by mixing them with biodegradable materials. Modifying the CPs backbone by adding typical enzymatically cleavable or hydrolysable linkages between biodegradable materials and CPs backbone also offers a scaffold with a higher biodegradability rate [104]. Table 4 shows a summary of the biodegradation trend of collagen, chitosan, and PEDOT: PSS-based scaffolds.

## 8. Current Developments of Polymeric Materials for Biomedical Applications

CPs hold favourable characteristics, such as electronic–ionic hybrid conductivity, mechanical softness, permeable porosity, and versatile chemical modification. This means they are recommended for a wide range of biomedical applications, including biosensors, chemical sensors, drug delivery systems, artificial muscles, and neural interfaces [105]. Additionally, CPs are utilized in the application of artificial muscles, due to their electrochemical deformation properties. The magnitude of the CPs’ strain depends upon their number of anions. Briefly, by applying a positive voltage with a suitable electrolyte, the polymer becomes oxidized, and the material loses electrons from the polymer. Then, a pair of anions are formed in the electrolyte. These anions cause the expansion of the polymer. The CP’s contraction mechanism (reduction reaction) is similar to the expansion mechanism (oxidation reaction) [106]. In addition, CPs can also be used to replace alkaline metal as biosensors, such as for non-enzymatic glucose sensors, hydrogen peroxide (H_2_O_2_) sensors, and dissolved oxygen sensors [107]. Recently, CPs like PANI was reported to have antibacterial properties through a disruption process against the native surface charge of bacterial cells [108]. This finding successfully proved that PANI can be utilized in producing antibacterial medical appliances. However, the use of CPs for skin biosensors was doubted, due to their biocompatibility issues (including inflammation and serious disorders) when in contact with living human physiology [109]. Hence, the utilisation of CP biomaterials in biomedical applications warrants more research in the near future, since CPs hold several drawbacks alongside their advantageous properties. Table 4 shows the current development of biodegradable natural and synthetic polymeric materials for various biomedical applications, including tissue engineering, temporary implants, wound healing, and drug delivery.

## 9. Conclusions

Fabrication techniques for conductive and biodegradable scaffolds could affect the physical and mechanical properties of the scaffold. Injectable scaffolds have the potential to be the most suitable low-risk method for TE-related medical treatments. Optimising the scaffold’s properties to resemble the properties of human ECM tissue is an ideal goal to realise a scaffold’s potential. This will lower the property gap between the scaffold and the tissues at the implantation site. Identical biodegradation and tissue regeneration rates are pivotal to ensure optimised healing. The by-products of scaffolds’ degradation should be non-toxic and biocompatible to inhibit immunogenic effects, particularly at the implantable site. In addition to the scaffold’s degradation rate, a proof of concept regarding biodegradation should be studied in vivo and in vitro. The biodegradability of CP-based scaffolds should be prioritised, without neglecting their electrical conductivity behaviour in future studies. The uses of CPs in biomedical applications are very broad, and each different CP has its own potential and speciality. Research, at the basic level, must be carried out, together with a focus on the varied aspects of study environment (in vitro and in vivo studies). This is because human bodies are very complex. Extensive studies focusing on the biocompatibility and immunogenicity of an electrically conductive scaffold composite must be conducted to obtain a clearer view of the use of CPs for tissue engineering applications.

## Figures and Tables

**Table 1:** Summary of scaffold fabrication techniques.

Fabrication Method	Scaffold Structure	Major Findings	Reference
Ionic grafting technique	Interconnected porous-hydrogels	Formation of conductive and self-healable hydrogels.	[29]
Lyophilization technique	Sponge scaffold	The mechanical properties of collagen sponges were improved with the addition of alginate. Future research can confirm its potency in the healing of skin ulcers.	[33]
Drop-casting technique	Hydrogel-patch scaffold	Stable and conductive scaffold patch.	[18]
Chemical mixing—a filtered technique	Hydrogel scaffold	Electroactive hydrogels with advantageous characteristics: covalently crosslinked porous 3D scaffolds with notable swelling ratio, excellent mechanical properties, electroactivity in physiological conditions and cell proliferation and differentiation.	[56]
Lyophilization technique	Porous-sponge scaffold	Scaffold biomimicry was enhanced with the addition of collagen. Collagen increases electrochemical impedance responses. Controlling scaffold’s mechanical properties is highly beneficial for understanding the factors influencing cell behaviour in 3D scaffold structures.	[30]
Electrospinning technique	Nanofibrous mat-structured scaffold	The application of PEDOT: PSS with special electrical and mechanical properties as a scaffold is recommended for cardiac TE.	[25]

**Table 2:** Mechanical and electrical properties of conductive-based scaffold materials.

Scaffold Material	Conductivity (S/m)	Mechanical Strength (MPa)	Major Findings	References
CS/nHAp/PEDOT sponges	9.72 ± 0.78 × 10^−6^	5.0–5.5	PEDOT: PSS/nHAp/CS is a promising scaffold, due to its porosity, microstructure, conductivity, and cell response.	[26]
Chitosan/aniline Patch	-	6.73–1.14	A better understanding of the role of conductive materials in electro-responsive tissues in ex vivo and in vivo models can be achieved by applying bioelectronic devices onto the biotic–abiotic interface.	[18]
CS/PEDOT: PSS nanofibrous	(1.5 × 10^−3^–7.63 × 10^−3^)	13.07 ± 1.09*–*18.78 ± 0.95	Scaffolds with PEDOT: PSS showed greater cell support without any cell toxicity. The smaller fibre diameter of the fibrous mat structure can aid cell attachment.	[25]
8% PEDOT-HA/Cs/Gel hydrogel	(3.16 × 10^−3^)	47.3 ± 0.3 × 10^−3^ (compressive)	An 8% PEDOT-HA/Cs/Gel hydrated scaffold, with the compressive modulus of 47.3 ± 0.3 × 10^−3^ MPa, is a viable candidate for brain tissue in nerve TE.	[9]
Conductive PEDOT layer assembled Cs/Gel	(6.51 × 10^−3^)—6th week (1.82 × 10^−3^)—8th week	-	Although the conductivity of scaffolds depreciated with progressing biodegradation, they still met the electrical conductivity requirements for electrical stimulation in neural TE application.	[10]

**Table 3:** Biodegradation index of various composite scaffolds.

Scaffold Composition	Scaffold’s Biodegradability Claims	Reference
PEDOT: PSS Chitosan Hydroxyapatite Nanoparticles	CS and nHAp/CS scaffolds showed approximately 30% weight loss after 1 month. PEDOT: PSS/nHAp/CS scaffold degraded with 10% weight loss. Incorporation of PEDOT: PSS enhanced the stability of scaffold in PBS.	[26]
PEDOT Hyaluronic acid Chitosan Gelatine	CS/Gel scaffold showed 86% weight loss after 8 weeks of incubation in lysozyme enzymatic solution. 10% PEDOT-HA/CS/Gel scaffold experienced 43% weight loss after 8 weeks of incubation. The presence of PEDOT and hyaluronic acid enhanced the stability and biodegradation resistance of scaffolds, due to low water permeability in hydrophobic PEDOT.	[9]
PEDOT Chitosan Gelatine Hyaluronic acid	Biodegradation of 0.5 wt.% PEDOT/CS/Gel scaffold was 72.55 ± 3.79% at week 8 in an enzymatic solution. Biodegradation of PEDOT/Cs/Gel scaffold was 59.97 ± 3.22%, while it was 47.15 ± 2.17% for PEDOT-HA/CS gel scaffold. The addition of PEDOT lowers the degradation rate of the scaffold. PEDOT nanoparticles mediated cell–surface interactions and enhanced cell’s growth and proliferation.	[41]
Chitosan PEDOT Gelatine	The conductivity of hydrated and dehydrated PEDOT/CS/Gel scaffold gradually decreased over the degradation period. Chitosan and gelatine gradually degraded in an enzymatic solution. The conductivity value of conductive scaffolds still met the required electrical value for neural tissue engineering application after 8 weeks of incubation time.	[10]
Gelatine Sodium Alginate PEDOT: PSS	Scaffolds showed 30% and 70% weight loss after 7 and 30 days of incubation in DMEM: F12 shaker incubator, respectively. There was no significant difference between scaffolds with or without a conductive-polymer.	[98]
Chitosan k-carrageenan N-isopropyl acrylamide (NIPAM)	Chitosan/k-carrageenan with 0 µL, 200 µL and 400 µL of gold (Au) recorded weight loss of 27.41%, 36.52%, and 40.12% in PBS solution at week 8, respectively. The addition of gold nanoparticles increased the weight loss due to decreasing the molecular weight of chitosan through the catalytic activity of Au. Hydrolysis, dissolution, and enzymatic cleavage are the types of degradation mechanisms involved.	[44]
Gellan PVA	PG-NFs scaffold was stable and can maintain its structure in PBS solution for up to 14 days of incubation. The initial weight loss is due to non-crosslinked gellan and PVA molecules.	[38]
Polycaprolactone (PCL) Polypyrrole-block- poly(caprolactone) (PPy-b-PCL)	PCL and PCL/PPy 2% scaffolds had degradation rates of 37.28% and 55.8%, respectively. The degradation rate of the scaffold in the incubator shaker increased with an increasing PPy-b-PCL concentration.	[68]
Hydroxyapatite Collagen I Chitosan Glutaraldehyde	Ha-Col-CS-GTA recorded 10% and 16% weight loss on day 1 and 21, respectively, after incubation for the biodegradation test using AF-MSCs. Scaffold without a crosslinker GTA showcased a higher degradation rate of 39% and 55% after day 1 and 21 of incubation time, respectively.	[13].
Polycaprolactone polypyrrole	The maximum weight loss of the PCL/chitosan mat was 20% in PBS solution after 14 days of incubation. An amount of 7.5% PPy in the PCL/chitosan mat scaffold showed 12% weight loss after incubation time. Adding PPy into the PCL/chitosan fibres slowed the weight loss and degradation rate.	[78].
Collagen Chitosan GTA Genipin TTP	The highest degradation rate (55.0 ± 3.78%) at day 21 and (62.0 ± 4.23%) 28 were recorded on the TPP crosslinked scaffold. The GTA solution crosslinked scaffold had the slowest degradation rate (17.7 ± 1.57% and 26.5 ± 2.98%) at day 21 and 28, respectively. The GTA vapour crosslink degradation rate was relatively higher (3.4 ± 2.85%) on day 28. The higher the crosslinking degree, the lower the degradation rate. GP and TPP were only able to crosslink 78.38 ± 8.20% and 143.27 ± 4.03%, respectively (crosslinking degree between the crosslinker and collagen/chitosan scaffolds).	[67].

**Table 4:** Polymeric materials for biomedical applications.

Specific Application	Polymer Type	Material	Reference
Drug delivery	Synthetic Biopolymer	poly(lactic-*co*-glycolic) acid (PLGA)	[110]
	Biodegradable natural polymer	chitosan	[111]
	Biodegradable natural polymer	kefiran	[112]
	Synthetic biopolymer	Poly (ethylene) glycol (PEG)	[113]
Tissue Engineering	Biodegradable natural polymer	gelatine	[94]
	Synthetic biopolymer	Polyurethane and modified polyurethane	[114]
	Biodegradable natural polymer	collagen	[115]
	Synthetic biopolymer	polyester derivatives, such as poly ɛ-caprolactone (PCL), polylactic acid (PLA), and polyglycolic acid (PGA)	[116]
Temporary Implants	Synthetic degradable polymer	shape memory polymers (with shape memory effects)	[117]
	Synthetic biopolymer	poly(lactic) acid (PLA)	[118]
Wound Healing	Biodegradable natural polymer	fibrinogen, hyaluronic acid, cellulose	[119]
	Synthetic polymer	polyvinyl alcohol	[120]
	Biodegradable natural polymer	protein derivatives, such as silk; collagen bacterial polyester, such as bacterial cellulose	[121]

## Data Availability

Not applicable.
